# A comprehensive analysis of adverse drug reactions in 2020–2023: case studies

**DOI:** 10.3389/fphar.2025.1628347

**Published:** 2025-10-02

**Authors:** Jinghui Zhai, Hongzhe Yan, Jinping Zhang, Huiyu Yan, Jie Ma, Sixi Zhang

**Affiliations:** ^1^ Department of Clinical Pharmacy, The First Hospital of Jilin University, Changchun, Jilin, China; ^2^ School of Pharmaceutical Science, Jilin University, Changchun, Jilin, China; ^3^ Division of Clinical Research, the First Hospital of Jilin University, Changchun, Jilin, China

**Keywords:** adverse drug reactions, severe adverse drug reactions, clinical factors, personalized treatment, anti-tumor drug

## Abstract

**Introduction:**

Adverse drug reactions (ADRs) have posed significant threats to patient safety and could potentially result in adverse clinical outcomes.

**Methods:**

In this study, we analyzed ADRs data reported by a large hospital spanning from 2020 to 2023, with a particular focus on identifying demographic and clinical factors associated with severe ADRs. The dataset encompassed 5,644 cases, incorporating variables such as patient age, gender, smoking and drinking history, allergies, medication type, and administration route.

**Results:**

Among these, 408 cases of severe ADRs underwent detailed examination. Additionally, the study delved into the correlation between adverse drug reactions symptoms (ADRS) and various drug types. According to research statistics, individuals in the middle-aged group (46–65 years) exhibited the highest proportion of severe ADRs at 36.77%. Females were significantly more affected than males, accounting for 66.67% of severe ADRs. Anti-tumor drugs emerged as the primary cause of severe ADRs, responsible for 52.70% of such incidents. Compared with other administration methods, intravenous injection was more prone to causing severe ADRs, with a likelihood of 53.92%. Furthermore, the blood system was identified as the ADRS where severe ADRs occurred at a significantly higher rate than other parts of the body, at 53.19%. Correlation analysis reveals a strong association between medication type and factors such as patient age, administration route, and ADRS. Notably, ADRS was also strongly linked to drug type, gender, and age.

**Conclusion:**

These findings collectively highlight the critical need for personalized treatment plans and targeted monitoring. Particular attention should be directed towards high-risk groups, such as middle-aged females and patients undergoing anti-tumor therapies. By doing so, it is possible to enhance drug safety and minimize the occurrence of severe ADRs.

## 1 Introduction

Adverse drug reactions (ADRs), defined as “noxious and unintended responses to a drug at doses normally used in humans” by the World Health Organization (WHO) in 1972 (Organization, 1972), remain a significant challenge in the healthcare industry. These ADRs are a leading cause of patients’ morbidity and mortality globally, placing substantial pressure on healthcare systems (Formica et al., 2018).

Europe study have found that 3.5% of hospitalizations are due to ADRs, and 10.1% of hospitalized patients experience ADRs during their stay (Bouvy et al., 2015). More broadly, studies have shown that approximately 16.9% of hospitalized patients experience ADRs (Rothschild et al., 2010), indicating their prevalence worldwide and the serious impact on patient safety (Lee and Chen, 2019; Bailey et al., 2016).

According to WHO standards, a severe ADR is defined as one that results in death, is life-threatening, requires hospitalization or extend the length of stay, causes disability or functional impairment, or has other medically important consequences. Though less common than ordinary ADRs, severe ADRs typically lead to longer hospital stays, additional treatment, and extended recovery periods (Hohl et al., 2011; Dalton and Byrne, 2017). These factors increase medical costs and significantly reduce the patients’ quality of life. Some studies have shown that serious ADRs accounted for 6.7% of hospitalizations and 0.3% of hospital mortality. These findings underscore the importance and urgency of accurately identifying and effectively managing severe ADRs to mitigate their impact on patients and healthcare systems.

Despite extensive international research on ADRs, particularly from the European Union and the United States, data provided by China, especially regarding hospitalized patients, remain limited (Song et al., 2023). There is relatively little research on the incidence and related factors of ADRs, especially severe ADRs, among hospitalized patients in China (Pitt et al., 2016).

To address this gap, we conducted a retrospective analysis of ADRs report data from a large hospital in China spanning 2020 to 2023. The northeastern region of China demonstrates a notably higher prevalence of chronic diseases like hypertension than the national average (Xing et al., 2020), a phenomenon intricately linked to climatic conditions, dietary patterns, and medical resource disparities. This unique convergence of factors not only elevates the complexity of ADRs in the region but also shapes a distinct epidemiological landscape where the cold climate exacerbates vascular stress, high-sodium/high-fat diets drive metabolic risks, and heterogeneous healthcare access influences medication practices. As a leading tertiary referral center, the First Hospital of Jilin University serves a diverse patient population spanning urban and rural areas of Northeast China, uniquely positioned to capture how these environmental, behavioral, and healthcare dynamics intermingle to impact ADRs profiles. The primary objectives were to describe the overall occurrence of ADRs and patient characteristics, and to investigate the association between ADRs severity and clinical factors. By focus on severe ADRs and analyzing their frequency, clinical manifestations, and potential risk factors, this study aims to provide valuable insights for improving medication safety in China’s medical environment.

## 2 Methods

### 2.1 Data source

This study used the ADRs data in the ADRs reporting system of a large hospital from 2020 to 2023, involving the demographic information of patients and details related to ADRs. All experimental protocols were approved by Ethics Committee of the First Hospital of Jilin University (No. 2023–567).

### 2.2 Data cleaning and extraction

Initial data cleaning was performed to extract valuable information, focusing on key variables such as age, gender, alcohol history, smoking history, allergy history, severity of adverse reactions, drug types, and route of administration.

### 2.3 Classifying of severe adverse reactions

The severity of ADRs is determined in accordance with the Reporting and Monitoring of Adverse Drug Reactions (Announced by the Ministry of Health on May 4, 2011 as Order No. 81. Effective from July 1, 2011).

### 2.4 Analysis of severe adverse reactions

For the analysis of severe adverse reactions, we incorporated each patient’s clinical manifestations to identify specific drug accumulation sites. This information was statistically analyzed to understand patterns and correlations related to the severity of the reactions (Tang and Lu, 2010; Emami Riedmaier et al., 2012).

### 2.5 Statistical analysis

Statistical analysis was conducted using the software available at SPSS Pro (Chan, 2004a; Chan, 2004b). Descriptive statistics summarized the characteristics of each group. A chi-square test was used to evaluate whether there was a significant association between categorical variables. When the χ^2^ test result is significant, it indicates that there is a statistical difference in the distribution of the two variables, that is, there is an association between them (but it does not mean causality) (Schober and Vetter, 2019; McHugh, 2013). All methods were carried out in accordance with relevant guidelines and regulations.

## 3 Results

### 3.1 Overall situation

Table 1 summarizes the demographic and clinical characteristics related to ADRs recorded from 2020 to 2023.

**TABLE 1:** Overall situation of ADRs.

Name	Option	Frequency	Percentage (%)	Cumulative percentage (%)
Age	<18	985	17.452	17.452
	19∼45	724	12.828	30.280
	46∼65	2,234	39.582	69.862
	66∼79	1,266	22.431	92.293
	>79	435	7.707	100
Sex	Female	3,627	64.263	64.263
	Male	2017	35.737	100
Drinking	No	5,140	91.07	91.07
	Yes	504	8.93	100
Smoking	No	5,347	94.738	94.738
	Yes	297	5.262	100
Allergic	No	5,466	96.846	96.846
	Yes	178	3.154	100
Ad	Intravenous drip	2,527	44.773	44.773
	other	1,556	27.569	72.342
	Oral administration	1,172	20.765	93.108
	Nebulization inhalation	287	5.085	98.193
	subcutaneous injection	102	1.807	100
Severity	commonly	5,236	92.771	92.771
	serious	408	7.229	100
Classes of drugs	Anti-tumor drugs	2006	35.542	35.542
	other	1,588	28.136	63.678
	Anti-infective drugs	866	15.344	79.022
	Respiratory system medication	553	9.798	88.82
	Circulating system drugs	398	7.052	95.872
	Hematological system drugs	233	4.128	100

The analysis of the ADRs data yielded several key insights. The age group most affected was 46–65 years old, accounting for 39.6% of cases. This indicates that middle-aged individuals may have a higher susceptibility to ADRs. Regarding gender distribution, females made up 64.3% of cases, compared to 35.7% for males. A notable 91.1% of cases has no history of drinking or smoking, suggesting that these factors were not primary contributors to the observed ADRs. Similarly, 96.9% of cases had no allergy history, which may imply that allergic reactions are less likely to be the main cause of ADRs in this dataset.

In terms of administration routes, intravenous (IV) injection was the most common at 44.8%, followed by oral administration at 20.8%. This highlights the need for healthcare personnel to closely monitor patients, especially during high-risk administration routes like IV (Wacker, 2013).

Most cases (92.8%) were non-severe, while 7.2% of cases were classified as severe. This aligns with previous relevant research findings. Anti-tumor drugs were the most common type involved, at 35.5% of cases. This underscores the potential risks associated with anti-tumor drugs and suggests a need to enhance surveillances of such drugs (Zhang et al., 2022).

### 3.2 Overall situation of serious adverse drug reactions

Table 2 categorizes the severe ADRs by age, sex, drinking history, allergy history, drug classes, smoking history, administration routes, and ADRS. The most affected age group for severe ADRs was 46–65 years, accounting for 36.8%. However, this age range represented 39.6% of the overall ADR group, indicating a slight decrease in the proportion of severe cases. The 66–79 years age group had a higher rate of severe reactions compared to their overall share, suggesting that individuals in this age range are more prone to severe ADRs. This emphasizes the need for heightened attention to medication safety in this older population (Zhang et al., 2020; Mangoni, 2012; Cacabelos et al., 2021).

**TABLE 2:** Overall situation of severe ADRs.

Name	Option	Frequency	Percentage (%)	Cumulative percentage (%)
Age	<18	139	34.069	70.833
	19∼45	40	9.804	96.078
	46∼65	150	36.765	36.765
	66∼79	63	15.441	86.275
	>79	16	3.922	100
Sex	Female	272	66.667	66.667
	Male	136	33.333	100
Drinking	No	381	93.382	93.382
	Yes	27	6.618	100
Smoking	No	389	95.343	95.343
	Yes	19	4.657	100
Allergic	No	403	98.775	98.775
	Yes	5	1.225	100
Classes of drugs	Anti-tumor drugs	238	58.333	58.333
	Other	84	20.588	78.922
	Anti-infectious agent	42	10.294	89.216
	Respiratory drugs	18	4.412	93.627
	Circulating system drugs	17	4.167	97.794
	Hematological system drugs	9	2.206	100
Ad	intravenous drip	220	53.922	53.922
	Other	149	36.52	90.441
	Oral	28	6.863	97.304
	Subcutaneous injection	9	2.206	99.51
	Nebulization inhalation	2	0.49	100
ADRS	Hematologic	217	53.186	53.186
	Mucocutaneous system	81	19.853	73.039
	Digestive system	31	7.598	80.637
	Other	28	6.863	87.5
	Systemic damage	21	5.147	92.647
	Respiratory system	17	4.167	96.814
	Cardiovascular system	13	3.186	100

In terms of sex distribution, females accounted for 66.7% of severe ADRs, compared to 64.3% in the overall ADRs group. This indicates that women are more likely to experience severe reactions, suggesting a higher risk of severe outcomes once an ADRs occurs. A large majority (93.4%) of severe ADRs had no history of drinking, and 95.3% had no smoking history, implying that alcohol and smoking have little significant effect on the occurrence of severe ADRs. Additionally, 98.8% of patients had no allergy history, further reinforcing the other factors, such as drug type or individual patient physiology, are more likely to influence the severity of reactions.

Anti-tumor drugs were the leading cause of severe ADRs, responsible for 58.3% of cases. Their high toxicity and narrow therapeutic window make them particularly dangerous, necessitating careful monitoring during treatment (Barachini et al., 2023; da Cunha de Medeiros et al., 2023; Pantazi and Tselepis, 2022; Curigliano et al., 2016).

IV administration was the most common route associated with severe ADRs, accounting for 53.9% of cases. This underlines the need for precise dosage control and monitoring due to the rapid and direct delivery of drugs via this method (Marsh et al., 2020; Chionh et al., 2017). Skin and mucous membranes were the next most affected areas, with 19.9% of severe cases.

The hematologic system was the most commonly affected in severe ADRs, representing 53.2% of cases. This indicates that if ADRs involve the hematologic system, there is a high probability they will be severe, thus requiring close monitoring.

### 3.3 Influence of drug types on the occurrence of severe ADRs

Table 3 demonstrates a significant association between drug category and several factors in severe ADRs. The Pearson chi-square test (χ ^2^ = 110.022, P = 0.000) revealed a highly significant association between age and drug category. Anti-tumor drugs were predominantly linked to severe ADRs in patients under 18 (118 of 139 cases) and those aged 46–65 (71 of 150 cases). This highlights the higher risk for young and middle-aged patients, particularly those undergoing chemotherapy. In contrast, other drug categories, such as anti-infective and cardiovascular drugs, showed a more even distribution of ADRs across different age groups. This underscores the importance of considering age-specific risks when prescribing these medications.

**TABLE 3:** Significance analysis of the influence of drug type on other factors of severe ADRs.

Contents		Anti-tumor drugs	Anti-infectious drugs	Circulating system drugs	Respiratory drugs	Blood system drugs	Others	Total	χ^2^	P
Age	<18	118	7	0	0	0	14	139	110.022	<0.0001
	19∼45	18	7	1	3	0	11	40		
	46∼65	71	13	10	9	5	42	150		
	66∼79	31	11	4	6	1	10	63		
	>80	0	4	2	0	3	7	16		
Ad	Intravenous drip	145	30	7	13	0	25	220	137.588	<0.0001
	Oral	6	0	4	2	4	12	28		
	Nebulization inhalation	0	0	0	2	0	0	2		
	Subcutaneous injection	6	0	0	0	2	1	9		
	Others	81	12	6	1	3	46	149		
ADRS	Hematologic	201	4	2	1	1	8	217	366.906	<0.0001
	Mucocutaneous system	4	31	3	12	1	30	81		
	Digestive system	18	2	4	0	3	4	31		
	Others	6	2	2	1	3	14	28		
	Systemic damage	5	2	0	0	1	13	21		
	Respiratory system	4	1	2	1	0	9	17		
	Cardiovascular system	0	0	4	3	0	6	13		

There was also a highly significant association between drug classes and Adverse Drug Reaction Symptoms (ADRS) (χ^2^ = 366.906, P < 0.0001). Anti-tumor drugs were mainly associated with hematologic system damage (201 of 238 cases), reflecting their known blood toxicity and emphasizing the need for close hematologic monitoring during chemotherapy. Conversely, anti-infective drugs were more likely to cause skin and mucosal reactions (31 of 42 cases), indicating the need for targeted dermatological monitoring for patients using these drugs. The clear association between different drug classes and specific accumulation sites should guide patient care strategies.

### 3.4 Characteristics of Adverse Drug Reaction Symptoms in severe ADRs

Table 4’s data analysis reveals significant relationships among age, gender, drug category, and ADRS. The Pearson chi-square test (χ^2^ = 106.246, P < 0.0001) shows a strong age-ADRS association. Patients under 18 are mainly affected by hematologic system damage (110 of 139 cases). In contrast, patients aged 46–65 show a wider range of ADRS, including the skin/mucosal system (36 cases), digestive system (15 cases), and general systemic damage (15 cases). This suggests younger patients are highly vulnerable to hematologic toxicity (Hardy-Abeloos et al., 2020; Teachey et al., 2022), while middle-aged adults face a broader ADRS. These findings highlight the need for age-specific monitoring: younger patients may need intensive hematologic evaluations, while middle-aged adults require comprehensive systemic assessments.

**TABLE 4:** Significance analysis of the characteristics of ADRS in severe ADRs.

Name	Option	ADRS							Total	Inspection method	χ^2^	P
		Hematologic	Mucocutaneous system	Digestive system	Systemic damage	Respiratory	Cardiovascular system	Other				
Age	<18	110	10	6	0	6	0	7	139	pearsonchi-square test	106.246	0.000***
	19∼45	12	10	9	2	3	2	2	40			
	46∼65	61	36	15	15	6	8	9	150			
	66∼79	31	20	1	3	1	2	5	63			
	>80	3	5	0	1	1	1	5	16			
Sex	Male	66	36	14	1	2	5	12	136	pearsonchi-square test	19.853	0.003***
	Female	151	45	17	20	15	8	16	272			
Classes of drugs	Anti-tumor	201	4	18	5	4	0	6	238	pearsonchi-square test	366.906	0.000***
	Anti-infectious agent	4	31	2	2	1	0	2	42			
	Circulating system drugs	2	3	4	0	2	4	2	17			
	Respiratory drugs	1	12	0	0	1	3	1	18			
	Blood system drugs	1	1	3	1	0	0	3	9			
	Other	8	30	4	13	9	6	14	84			

* * *, * * *, and * represent 1%, 5%, and 10% significance levels, respectively.

There’s also a significant sex-ADRS (χ^2^ = 19.853, P = 0.003). Males are more prone to hematologic reactions (66 of 136 cases), while females tend to have a wider reaction range, especially skin/mucosal system (45 cases) and general systemic damage (20 cases). This implies females may be more susceptible to dermatologic and systemic reactions. These results stress the importance of sex-specific clinical monitoring, particularly for females more prone to severe skin and mucosal reactions.

Moreover, drug classes and ADRS are strongly associated (χ^2^ = 366.906, P < 0.0001). Anti-tumor drugs are predominantly linked to hematologic damage (201 of 238 cases), reflecting their known blood toxicity. In contrast, anti-infective drugs are more often associated with skin and mucosal reactions (31 of 42 cases).

These results emphasize the need for tailored monitoring based on drug class. Patients undergoing chemotherapy require close hematologic evaluation, while those on anti-infective drugs need vigilant dermatologic monitoring. This approach can mitigate drug-class-specific risks and reduce severe ADRs likelihood.

## 4 Discussion

**FIGURE 1:**
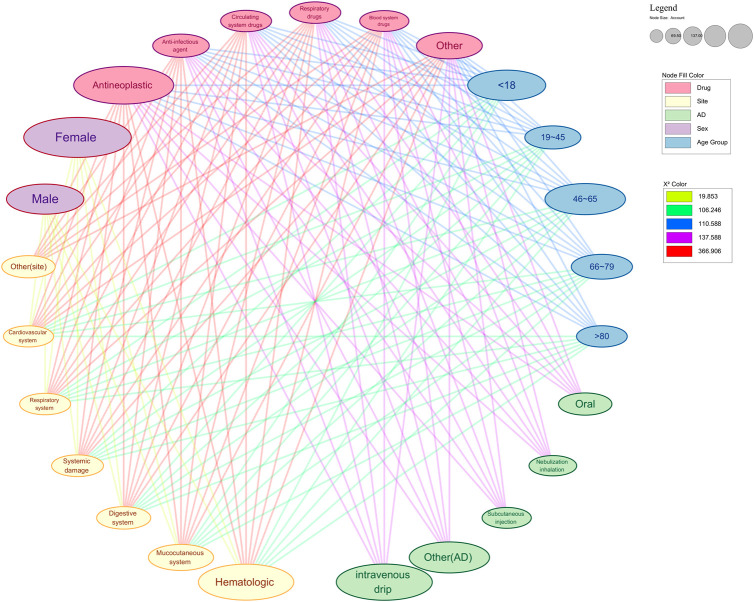
Network diagram of significant analysis results of various factors of serious ARDs. This figure shows the significance analysis results with drug type and drug accumulation site as independent variables. The color of the nodes in the graph represents the group they belong to. The color of the edge reflects the strength of saliency. Nodes connected to each other imply a significant correlation and mutual influence between the two. AD: administration.

Our analysis of ADRs from 2020 to 2023 reveals the complex patterns of severe ADRs, with a focus on demographic and clinical factors. Middle-aged adults (46–65 years old)are the most affected, comprising 39.58% of all ADRs and 36.77% of severe cases. This heightened vulnerability may stem from increased health issues, polypharmacy, and age-related alterations in drug metabolism (Budnitz et al., 2021; Cardelli et al., 2012; Ragoonanan et al., 2021; Figure 1).

The study also shows a higher incidence of ADRs in women, who account for 64.26% of all cases and 66.67% of severe ADRs. This suggests that gender differences in drug metabolism and response are significant, underscoring the need to consider biological and social factors when assessing patient risks. Factors such as body composition, hormonal differences, and healthcare access may contribute to women’s increased risk of severe reactions (Moyer et al., 2019; de Vries et al., 2019). It was reported that gender significantly influences drug metabolism through sex-specific differences in cytochrome P450 enzyme activities (e.g., higher CYP3A4 activity in females and CYP1A2/CYP2E1 in males), hormonal regulation (estrogen and testosterone modulating glucuronidation and CYP expression), and pharmacokinetics (absorption, distribution, and renal excretion) (Scandlyn et al., 2008). These mechanisms lead to clinical implications such as gender-specific dosing requirements and varying rates of drug-induced toxicity (Schmetzer and Flörcken, 2012).

A notable finding is the high rate of ADRs associated with IV administration, which accounts for 53.92% of severe ADRs. The rapid delivery of drugs via IV can lead to immediate reactions, particularly with potent agents like anti-tumor drugs. This emphasizes the need for vigilant monitoring during IV therapy, especially for chemotherapy patients who are already at a higher risk of severe ADRs (Wacker, 2013).

Interestingly, the study also establishes links between drug classes and ADRS, helping to pay attention to the occurrence of ADRs. Younger patients, particularly those under 18, show a higher incidence of blood-related ADRs, highlighting the need for careful hematologic monitoring in this group. In contrast, middle-aged adults experience a broader range of reactions, likely due to their underlying health conditions and the multiple medications they take.

While our findings are valuable, we must acknowledge several limitations. The study is based on passive surveillance data from a hospital reporting system, which inherently suffers from underreporting and reporting bias due to its reliance on voluntary submissions. Mild or non-life-threatening ADRs are likely underrepresented, while severe ADRs may be overemphasized, skewing the observed distribution of ADRs severity and demographic associations. For example, the lower proportion of elderly individuals (80+) in severe ADRs might not reflect their true risk but rather underreporting due to under recognition or under documentation of ADRs in clinical settings. Additionally, the retrospective design may introduce biases from missing data, unaccounted comorbidities, and drug interactions, potentially affecting the accuracy of our analyses. The focus on specific age and gender groups also risks overlooking other at-risk populations. Reporting bias further arises from variations in healthcare-seeking behaviors or institutional reporting practices, introducing systematic errors in data collection. These limitations mean our results may not fully capture real-world ADR prevalence, especially among underrepresented groups. When interpreting findings, it is critical to recognize that observed associations could be influenced by incomplete data, and conclusions about risk profiles require cautious extrapolation to broader populations.

Future research should adopt a longitudinal approach to investigate the effects of high-risk drugs, particularly anti-tumor drugs, across more diverse patient populations. Exploring genetic and physical factors influencing drug metabolism could enhance our understanding of individual-level risks. By implementing personalized treatment and monitoring strategies that account for patient demographics, drug types, and administration methods, we can improve medication safety and patients’ outcomes.

## 5 Conclusion

The occurrence of ADRs is significantly influenced by various demographic and clinical factors, with middle-aged adults, female, individuals undergoing intravenous drug administration, and those on anti-tumor drugs facing elevated risks. Recognizing the complex interplay of these factors in severe ADRs is essential for enhancing the efficacy of monitoring practices and developing personalized treatments strategies. This approach can ultimately improve patient safety and clinical outcomes.

## Data Availability

The original contributions presented in the study are included in the article/supplementary material, further inquiries can be directed to the corresponding author.
